# Comparison of the porcine uterine smooth muscle contractility on days 12–14 of the estrous cycle and pregnancy

**DOI:** 10.1186/s13028-016-0201-z

**Published:** 2016-03-22

**Authors:** Włodzimierz Markiewicz, Marek Bogacki, Michał Blitek, Jerzy Jan Jaroszewski

**Affiliations:** 1 Department of Pharmacology and Toxicology, Faculty of Veterinary Medicine, University of Warmia and Mazury, Oczapowskiego Street 13, 10-718 Olsztyn, Poland; 2 Institute of Animal Reproduction and Food Research, Polish Academy of Sciences, Bydgoska Street 7, 10-243 Olsztyn, Poland

**Keywords:** Early pregnancy, Embryos, Gilts, Uterine contractility

## Abstract

**Background:**

Uterine contractile activity is very important for many reproductive functions including embryo transport, implantation, gestation and parturition. Abnormal contractility leads to implantation failure, spontaneous miscarriage, preterm birth and many other disorders. The objective of the present study was to assess the effects of acetylcholine (ACh), noradrenaline (NA), oxytocin (OT) and prostaglandins F_2α_ (PGF_2α_) and E_2_ (PGE_2_) on the contraction of uterine strips collected from the horns of cyclic gilts (12–14 days of the estrous cycle—group I) and from pregnant (12–14 days after first insemination gilts in which one of the uterine horn was gravid (group IIa) and the second one was non-gravid (group IIb). Uterine strips consisting of the endometrium with the myometrium and myometrium alone were examined.

**Results:**

ACh increased the tension in all groups as compared to the pretreatment period, and the increase was the highest in group IIb; the amplitude decreased in all groups, and the frequency increased mainly in groups I and IIa. NA did not affect the tension in any group, but decreased the amplitude and frequency in group IIb as compared to groups I and IIa. OT caused the highest increase in the tension in group IIb, a decrease in the amplitude and an increase in the frequency of contractions as compared to the pretreatment period. PGF_2α_ induced the highest increase in the tension and amplitude in group IIb, with a decline in the frequency in this group. PGE_2_ increased the tension and frequency only in group IIb, and caused the greatest eduction in the amplitude in this group.

**Conclusions:**

These results indicate that contractility of the porcine smooth muscle collected from uterine horns with embryos was different from those obtained from the uterine horns without embryos and the horns of cyclic gilts.

## Background

It is generally acknowledged that uterine contractility is regulated by complex interactions between many factors. Contractions determine the motor activity of the uterus which is particularly important during the migration of embryos and its implantation in the uterus. Contractions and relaxations of the porcine myometrium are controlled by the autonomic nerve system [1]. Moreover many endocrine and auto/paracrine/factors are also involved in this regulation [2–4]. Uterine contractions in pigs can be stimulated by compounds such as acetylcholine (ACh) [5], oxytocin (OT) [6–8], prostaglandins F_2α_ (PGF_2α_) [7–9] and E_2_ (PGE_2_) [7, 8, 10], histamine [11], neuropeptide Y (NPY) [12], and endothelin [13]. On the other hand, the relaxation of the porcine myometrium may be caused by noradrenaline (NA), serotonin (5-HT) [14], and nitric oxide [15]. These factors influence smooth muscle contractility directly or indirectly by affecting the synthesis and release of other substances. It has been shown that both exogenous and endogenous ACh cause contraction of the myometrium through the activation of the muscarinic M_3_ receptor [5]. Other data have shown that NA causes an excitatory response, predominantly via α_2_-adrenergic receptors, and stimulation of β_2_-adrenergic receptors inhibits contractile activity of the porcine myometrium [1]. It is also generally accepted that OT stimulates uterine contractile activity via its receptors [6], which are present in the porcine uterus [16]. Oxytocin, acting through its receptors in the endometrium and myometrium, is involved in the control of PGF_2α_ and PGE_2_ secretion in pigs [17, 18]. Prostaglandin F_2α_ contracts the uterine muscle indirectly by enhancing the responsiveness to OT followed by the prostaglandin PGF_2_ receptor (FP) mediated regression of the corpora lutea (decrease in plasma progesterone levels) [2]. However, the direct action of PGF_2α_ on contractile (FP, EP_1_, EP_3_) and relaxatory (DP, IP, EP_2_) receptors in porcine uterine smooth muscle has also been described [2]. Similarly, PGE_2_ may cause contraction or relaxation through its impact on particular EP receptor subtypes [10].

In gilts, myometrial activity undergoes changes during the oestrous cycle [2, 9, 10, 14, 19], at the time of mating and insemination [20, 21], and during the course of pregnancy [3, 22]. The early pregnancy in the pig is divided into three periods: post-conception (days 1–10 of pregnancy), the maternal recognition of pregnancy (days 11–13) and implantation (days 14–19) [23]. Thus, the period between the 12th and 14th days of the pregnancy is crucial for the successful implantation. The effects of various substances on motor activity of non-pregnant and pregnant porcine uteri have been studied, however, a comparison of the contractile activity between 12–14 days of the estrous cycle and pregnancy has not yet been made, especially in regard to the presence or not of embryos in the uterine horns. Therefore, the aim of our study was to examine the effects of ACh, OT, PGF_2α_, and PGE_2_ on the contraction of uterine strips collected from the horns of cyclic gilts and from the horns with and without embryos of early pregnant pigs. Selection of the substances was made on their importance in the regulation of reproductive processes and the endogenous activity in the reproductive tract. The receptor mechanisms of these substances are also used in the case of drugs that interfere with the processes of uterine contractility. Until now, the differences in the impact of tested substances within the a period that is a crucial for a successful implantation compared to the analogous period of the estrous cycle are unknown. Therefore, we hypothesize that the uterine contractile response to stimuli will be affected by the presence of embryos within the uterus.

## Methods

### Animals

Prepubertal crossbred gilts (n = 10) with a body weight of 106 ± 4.8 kg and approximately 7 months of age were used. The gilts were subjected to surgical procedure under general anaesthesia. The animals were premedicated with azaperone (2 mg/kg bw i.m.; Stresnil, Janssen Animal Health, Belgium) and ketamine (12 mg/kg bw i.m.; VetaKetam, Vet-Agro, Poland), and anaesthetized with thiopental (20–30 mg/kg bw i.v.; Thiopental, Sandoz, GMBH, Austria). In five gilts one of the uterine horns was separated according to a surgically-generated model described previously by [24]. Briefly, the uterus was presented by a midventral opening of the caudal part of the abdomen. Thereafter, one horn was cut transversely and the ends were closed by a suture. This way the uterus consisted of one whole uterine horn and a part of the second horn, both connected with the uterine corpus. The remaining part of the second horn, connected with the contiguous ovary, was surgically detached from the uterine corpus. Ten days after surgery gilts were treated hormonally by an intramuscular injection of 750 I.U. of eCG (Folligon, Intervet, Poland) and 500 I.U. of hCG (Chorulon, Intervet) given 72 h later. Subsequently, 24 h after the hCG treatment the gilts were inseminated twice at 12 h intervals. Only gilts with the symptoms of heat were used for the next stages of procedures. Heat were checked by observation of symptoms: sticky discharge from vulva, clitoris red and protruding and the standing behaviour after applying pressure on the back and flanks of the gilt. The remaining five gilts were treated hormonally in the same way but they were not inseminated. On days 12–14 after insemination or for non-bred animals the gilts were slaughtered. To confirm pregnancy, the uterine horns were flushed with 10 ml PBS to determine the presence of embryos in uterine flushings [24]. The exact number of embryos was not possible to count because of their defragmentation caused by flushing. All procedures involving animals were conducted in accordance with the rules approved by the Local Ethics Commission of the University of Warmia and Mazury in Olsztyn.

### Preparation of the uterine strips and measurements of their contraction

Fragments of the uterine horns, collected from the middle part of the horns, were transferred to ice, moved to the laboratory and immediately processed for examination of contractile activity. Uterine tissue was collected from the horns of cyclic gilts (group I) and the horns of pregnant gilts without (group IIa) and with embryos (group IIb). The contractile activity was examined according to the method described previously [4, 19]. Briefly, two kinds of the uterine strips (3 × 5 mm) consisting of the endometrium with myometrium (ENDO/MYO) and myometrium (MYO) alone were resected. The study involved these two types of strips because in previous studies we found that the presence of endometrium affected the contractile activity of myometrium [20]. From each examined horn of the uterus two ENDO/MYO and two MYO strips were selected. After resection the strips were washed in saline and mounted between two stainless steel hooks in 5 ml of an organ bath (Schuler Organ bath type 809; Hugo Sachs Electronic, Germany) under conditions of resting tension of 5 mN. The strips were kept in the Krebs-Ringer solution of the following composition (mM/l): NaCl, 120.3; KCl, 5.9; CaCl_2,_ 2.5; MCl_2,_ 1.2; NaHCO_3,_ 15.5; glucose, 11.5; 37 °C, pH 7.4. The solution was maintained at 37 °C and continuously saturated with a mixture of 95 O_2_ and 5 % CO_2_. Measurements of smooth muscle contraction were conducted using a force transducer (HSE F-30 type 372), and a bridge coupler type 570, while the graphic recording was made on a recorder (Hugo Sachs Elektronik) with HSE-ACAD W software.

### Schedule of contractile activity examination

The recording was started after prior equilibration for at least 60 min. Thereafter, the strips were incubated with ACh (10^−5^–10^−4^ M; Sigma, St. Louis, MO, USA), NA (10^−7^–10^−6^ M; Levonor Polfa, Poland), OT (10^−7^–10^−6^ M, Vet-Agro, Poland), PGF_2α_ (10^−8^–10^−7^ M; Sigma), and PGE_2_ (10^−8^–10^−7^ M; Sigma). The doses of the substances tested were based on previous studies [9, 10, 19, 22]. Contractile activity was measured for 10 min after the administration of each concentration of the examined substance. At the end of the examination of each substance tissue chambers were washed three times with 15 ml of Krebs-Ringer solution at 10 min intervals. Finally, at the end of treatment with examined substances to determine the viability of tissues, ACh was repeatedly administered in the same doses as given before. Only those results for which the difference in response to the stimulation by ACh at the beginning and the end of the treatment were less than 20 % were included into the statistical analysis.

### Statistical analysis

Numerical values of the contractile activity (intensity, amplitude and frequency) of the strips before the application of the examined substances were calculated for 10 min and accepted as 100 %. The results calculated for 10-min periods after treatments were expressed as a percentage (mean ± SD) of the contraction intensity, amplitude and frequency before drug administration. The statistical significance of the differences was assessed by one-way analysis of variance ANOVA (Graphpad PRISM 3.1; Graphpad Software, San Diego, CA, USA), followed by Bonferroni’s multiple comparison test. Differences at *P* < 0.05 were considered statistically significant.

## Results

**Fig. 1:**
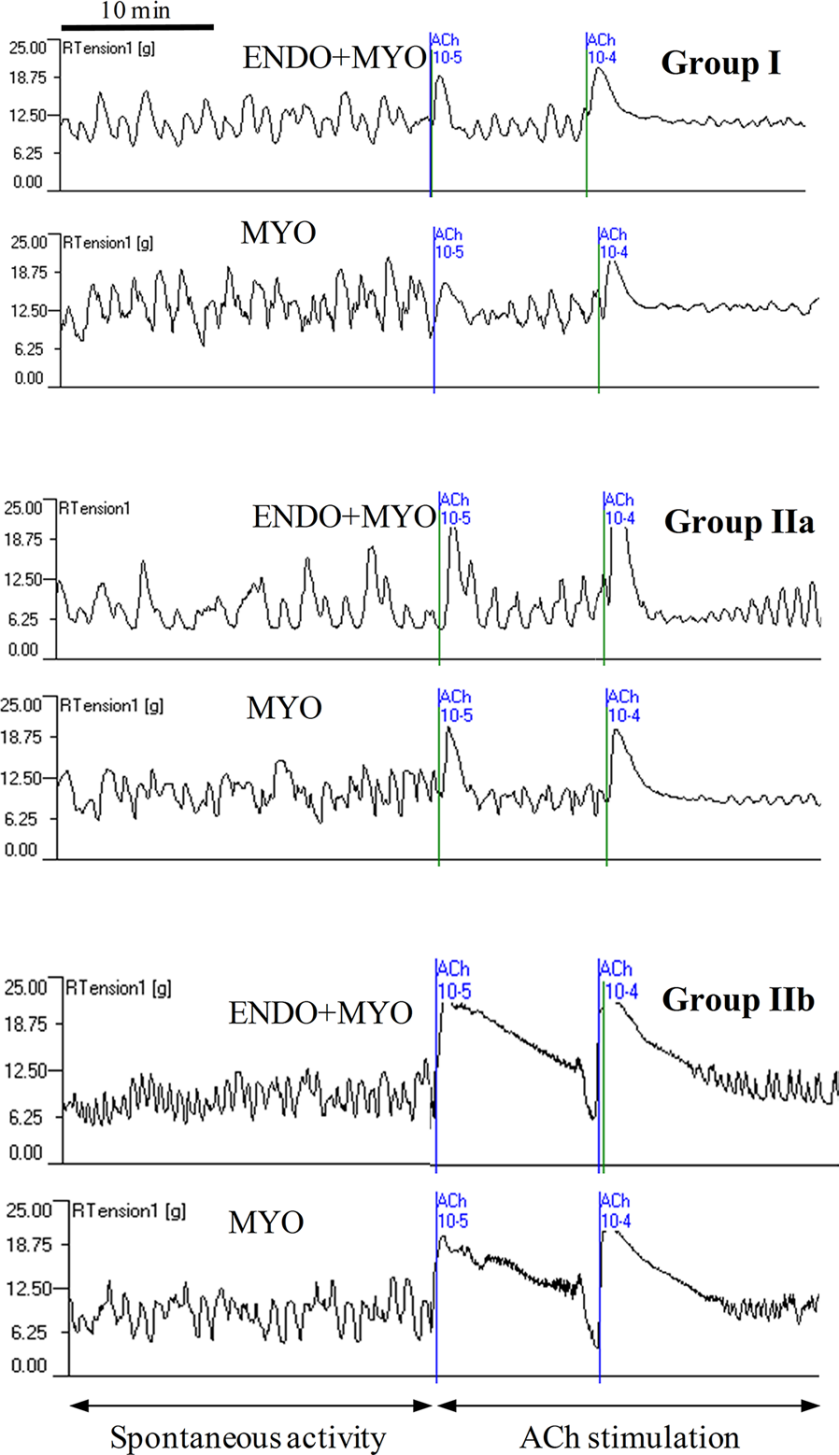
A representative diagram showing spontaneous contractile activity and motor activity after acetylcholine (ACh) administration at doses 10^−5^ and 10^−4^ M in the endometrium/myometrium (ENDO+MYO) and myometrium (MYO) strips collected on days 12–14 of the oestrous cycle (Group I) or after first insemination (groups without embryos—Group IIa and with embryos—Group IIb)

The spontaneous contractile activity before ACh administration in all examined tissues is shown in Fig. 1. Analysis of this activity did not demonstrate statistically significant differences between groups. Therefore, in the further statistical analysis the changes in the contractile activity after administration of the tested substances were compared to the pretreatment period.

### Influence of ACh on uterine contractile activity

**Fig. 2:**
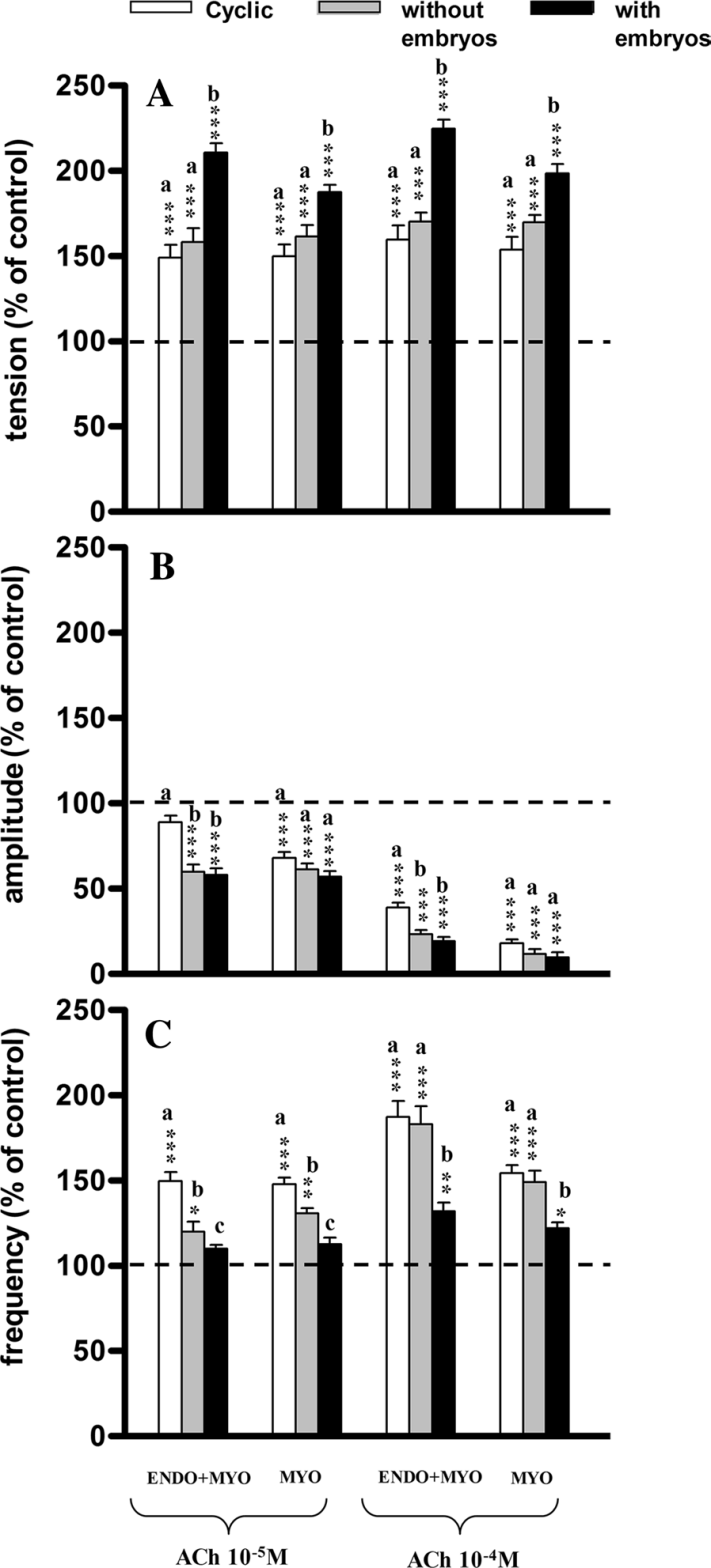
Influence of acetylcholine (ACh) on the tension (**A**) amplitude (**B**) and frequency (**C**) of the contraction of the porcine endometrium/myometrium (ENDO+MYO) and myometrium (MYO) strips collected on days 12–14 of the oestrous cycle (cyclic) or after first insemination) (groups without embryos and with embryos). Values (mean ± SD) represents five uterine horns examined in two strips from each one and are expressed as a percentage of changes in the contractile activity before the treatment. *P < 0.05, **P < 0.01, ***P < 0.001 as compared to the contractile activity before the treatment; *a*, *b*, *c* indicates differences between groups (cyclic, without embryos and with embryos)

The administration of ACh in both doses caused a significant (*P* < 0.001) increase in the tension in all groups as compared to the pretreatment period (Fig. 2A). The magnitude of the increase was greater (*P* < 0.01−*P* < 0.001) for strips from gravid horns than from non-gravid horns or cyclic gilt strips.

Administration of ACh caused a significant (*P* < 0.001) decrease in the amplitude in all groups (except ENDO/MYO in group I at a concentration of 10^−5^ M) as compared to the pretreatment period (Fig. 2B). Moreover, significantly smaller changes in the amplitude of the ENDO/MYO strips were determined in group I as compared to groups IIa and IIb (*P* < 0.001 after a dose of 10^−5^ M and *P* < 0.05 after a higher dose); such differences were not observed in the MYO strips.

The frequency of contractions in ENDO/MYO and MYO increased significantly (*P* < 0.05−*P* < 0.001) after the application of ACh at both doses in groups I and IIa as compared to the pretreatment period (Fig. 2C). In group IIb a significant increase (*P* < 0.05−*P* < 0.01) was observed only after the higher dose of ACh. After the lower dose of ACh the increase in frequency of contractions was significantly higher in the ENDO/MYO of group I as compared to groups IIa and IIb (*P* < 0.001), and in group IIa as compared to group IIb (*P* < 0.05); also in MYO strips a significantly greater increase was observed in group I as compared to groups IIa (*P* < 0.01) and IIb (*P* < 0.001), and in group IIa as compared to group IIb (*P* < 0.05). After the higher dose of ACh there were no differences between groups I and IIa, while a significantly (*P* < 0.05−*P* < 0.001) lower increase in group IIb was observed in both kind of strips as compared to groups I and IIa.

### Influence of NA on uterine contractile activity

**Fig. 3:**
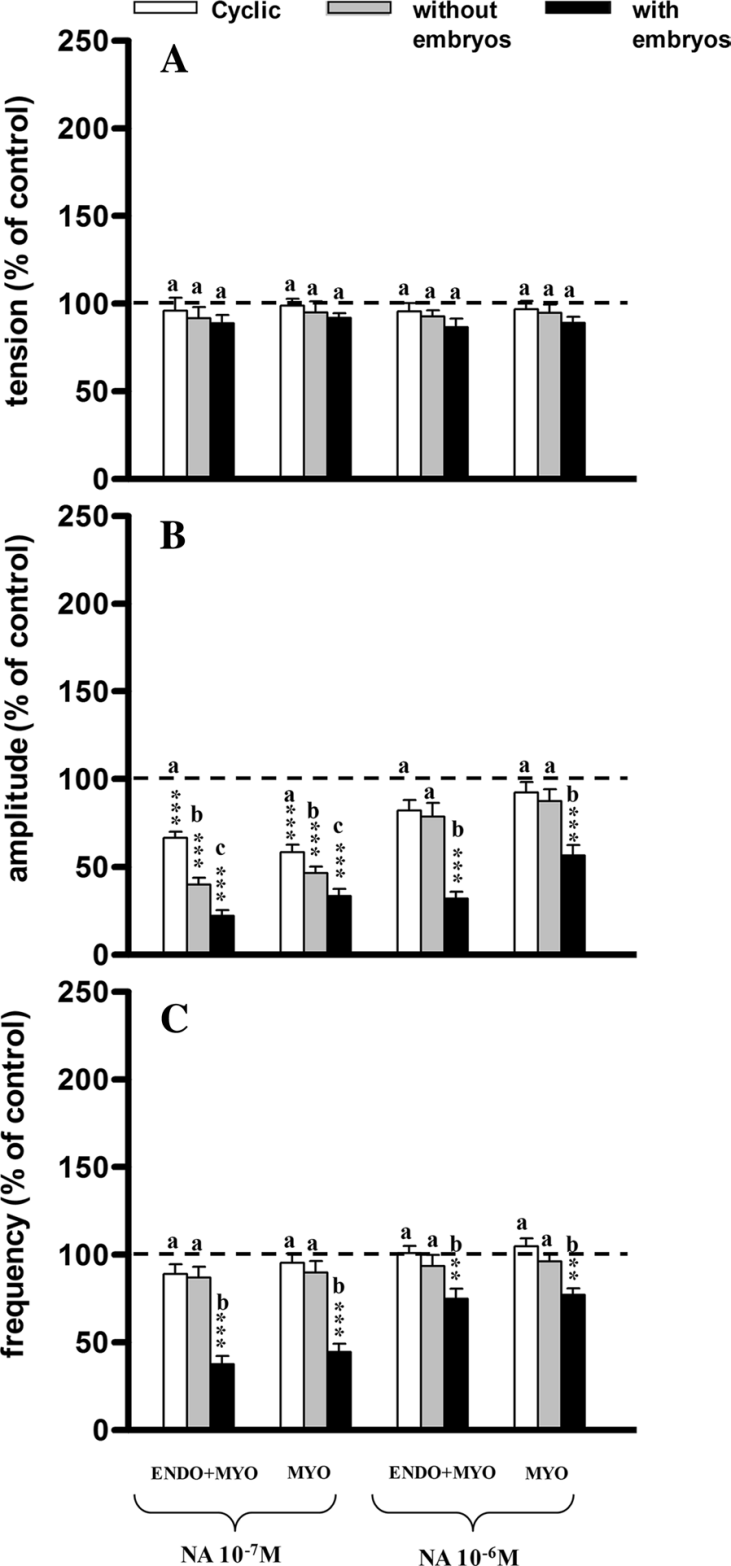
Influence of acetylcholine (NA) on the tension (**A**) amplitude (**B**) and frequency (**C**) of the contraction of the porcine endometrium/myometrium (ENDO+MYO) and myometrium (MYO) strips collected on days 12–14 of the oestrous cycle (cyclic) or after first insemination) (groups without embryos and with embryos). Values (mean ± SD) represents five uterine horns examined in two strips from each one and are expressed as a percentage of changes in the contractile activity before the treatment. **P < 0.01, ***P < 0.001 as compared to the contractile activity before the treatment; *a*, *b*, *c* indicates differences between groups (cyclic, without embryos and with embryos)

Administration of NA at both doses insignificantly influenced tension as compared to the pretreatment period (Fig. 3A); such differences were also not observed between the experimental groups.

The amplitude of contractions was significantly (*P* < 0.001) decreased after both doses in the ENDO/MYO and MYO in group IIb, and only after the smaller dose in groups I and IIa as compared to the pretreatment period (Fig. 3B). After NA administration in a dose of 10^−7^M significantly lower amplitude was observed in group IIb as compared to group I (*P* < 0.001), but this change was less evident as compared to group IIa (*P* < 0.05); significantly lower (*P* < 0.05−*P* < 0.01) amplitude was also observed in group IIa as compared to group I in both kinds of the strips.

The frequency of contractions decreased significantly (*P* < 0.01−*P* < 0.001) only in group IIb as compared to the pretreatment period (Fig. 3C), as well as compared to groups I and IIa.

### Influence of OT on uterine contractile activity

**Fig. 4:**
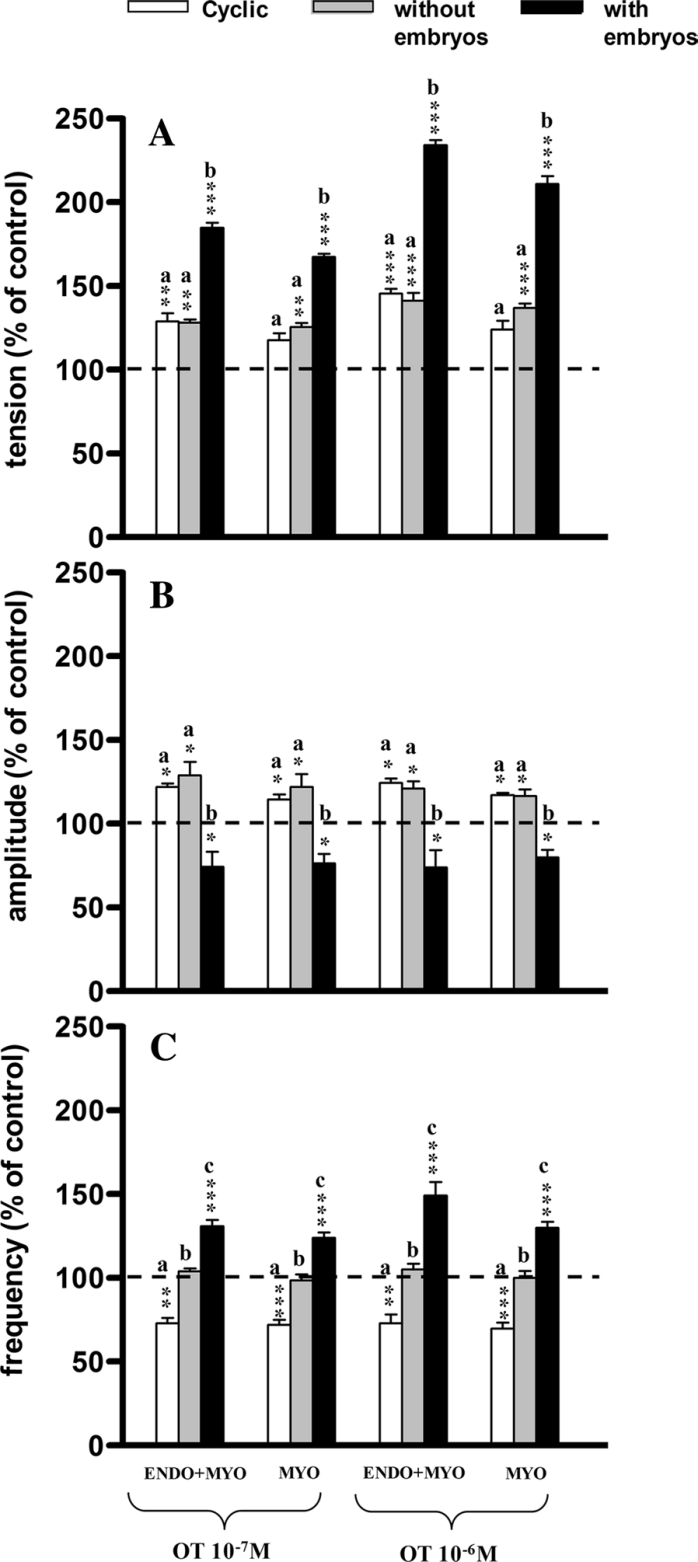
Influence of oxytocin (OT) on the tension (**A**) amplitude (**B**) and frequency (**C**) of the contraction of the porcine endometrium/myometrium (ENDO+MYO) and myometrium (MYO) strips collected on days 12–14 of the oestrous cycle (cyclic) or after first insemination) (groups without embryos and with embryos). Values (mean ± SD) represents five uterine horns examined in two strips from each one) and are expressed as a percentage of changes in the contractile activity before the treatment. *P < 0.05, **P < 0.01, ***P < 0.001 as compared to the contractile activity before the treatment; *a*, *b*, *c* indicates differences between groups (cyclic, without embryos and with embryos)

In the ENDO/MYO strips OT at both doses and in all groups caused a significant (*P* < 0.01−*P* < 0.001) increase in tension as compared to the pretreatment period (Fig. 4A); a significant increase was also observed in the MYO strips in groups IIa and IIb. Insignificant differences (*P* > 0.05) were noticed in tension in both ENDO/MYO and MYO strips between groups I and IIa, while significantly higher (*P* < 0.001) tension was found in group IIb as compared to groups I and IIa.

A significant (*P* < 0.05) increase in amplitude was observed after OT application at both doses in the ENDO/MYO and MYO strips in groups I and IIa, with a significant (*P* < 0.05) decrease in group IIb as compared to the pretreatment period (Fig. 4B). Significantly (*P* < 0.001) lower amplitude was also determined between group IIb as compared to groups I and IIa in both kinds of strip.

In ENDO/MYO and MYO strips the frequency of contractions decreased significantly in group I (*P* < 0.01−*P* < 0.001) but increased in group IIb (*P* < 0.001), and was not changed in group IIa as compared to the pretreatment period (Fig. 4C). The frequency of contractions was significantly lower in group I as compared to groups IIa (*P* < 0.01) and IIb (*P* < 0.001), and in group IIa as compared to group IIb (*P* < 0.01−*P* < 0.001).

### Influence of PGF_2α_ on uterine contractile activity

**Fig. 5:**
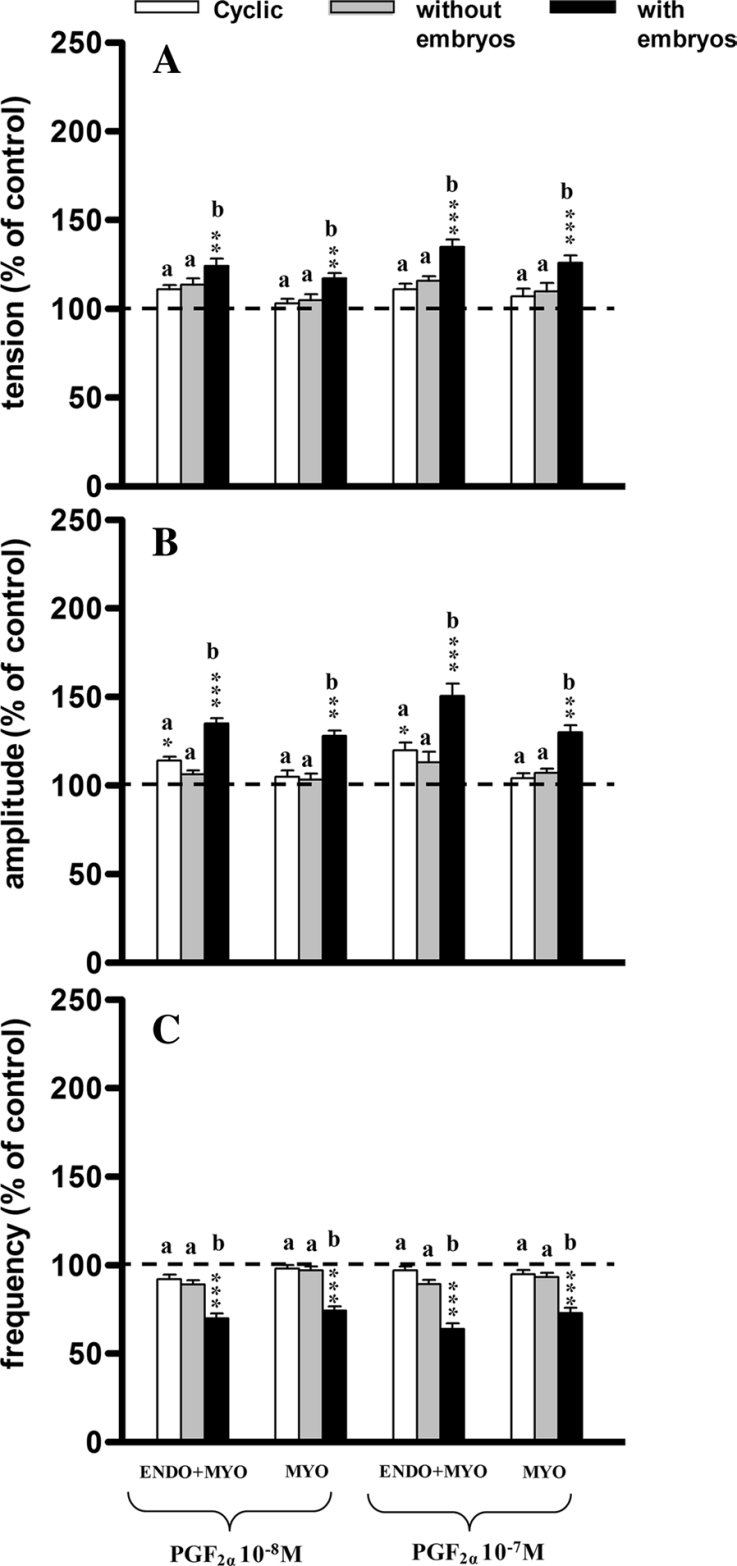
Influence of prostaglandin F_2α_ (PGF_2α_) on the tension (**A**) amplitude (**B**) and frequency (**C**) of the contraction of the porcine endometrium/myometrium (ENDO+MYO) and myometrium (MYO) strips collected on days 12–14 of the oestrous cycle (cyclic) or after first insemination) (groups without embryos and with embryos). Values (mean ± SD) represents five uterine horns examined in two strips from each one and are expressed as a percentage of changes in the contractile activity before the treatment. *P < 0.05, **P < 0.01, ***P < 0.001 as compared to the contractile activity before the treatment; *a*, *b*, *c* indicates differences between groups (cyclic, without embryos and with embryos)

In both kinds of strip a significant increase (*P* < 0.01−*P* < 0.001) in tension after PGF_2α_ administration was observed only in group IIb as compared to the pretreatment period (Fig. 5A). There were no significant changes in tension between groups I and IIa, while significantly higher tension (*P* < 0.05−*P* < 0.01) was observed in group IIb as compared to groups I and IIa.

PGF_2α_ significantly increased the amplitude in the ENDO/MYO strips in groups I (*P* < 0.05) and IIb (*P* < 0.001), and in the MYO strips in group IIb (*P* < 0.01) as compared to the pretreatment period (Fig. 5B). There were no significant changes in amplitude between groups I and IIa, while significantly higher (*P* < 0.01−*P* < 0.001) amplitude was observed in group IIb as compared to groups I and IIa.

In the ENDO/MYO and MYO strips the frequency of contractions after PGF_2α_ administration decreased significantly (*P* < 0.001) in group IIb as compared to the pretreatment period (Fig. 5C). There were no significant changes in the frequency of contractions between groups I and IIa, while significantly (*P* < 0.01−*P* < 0.001) lower frequency was observed in group IIb as compared to groups I and IIa.

### Influence of PGE_2_ on uterine contractile activity

**Fig. 6:**
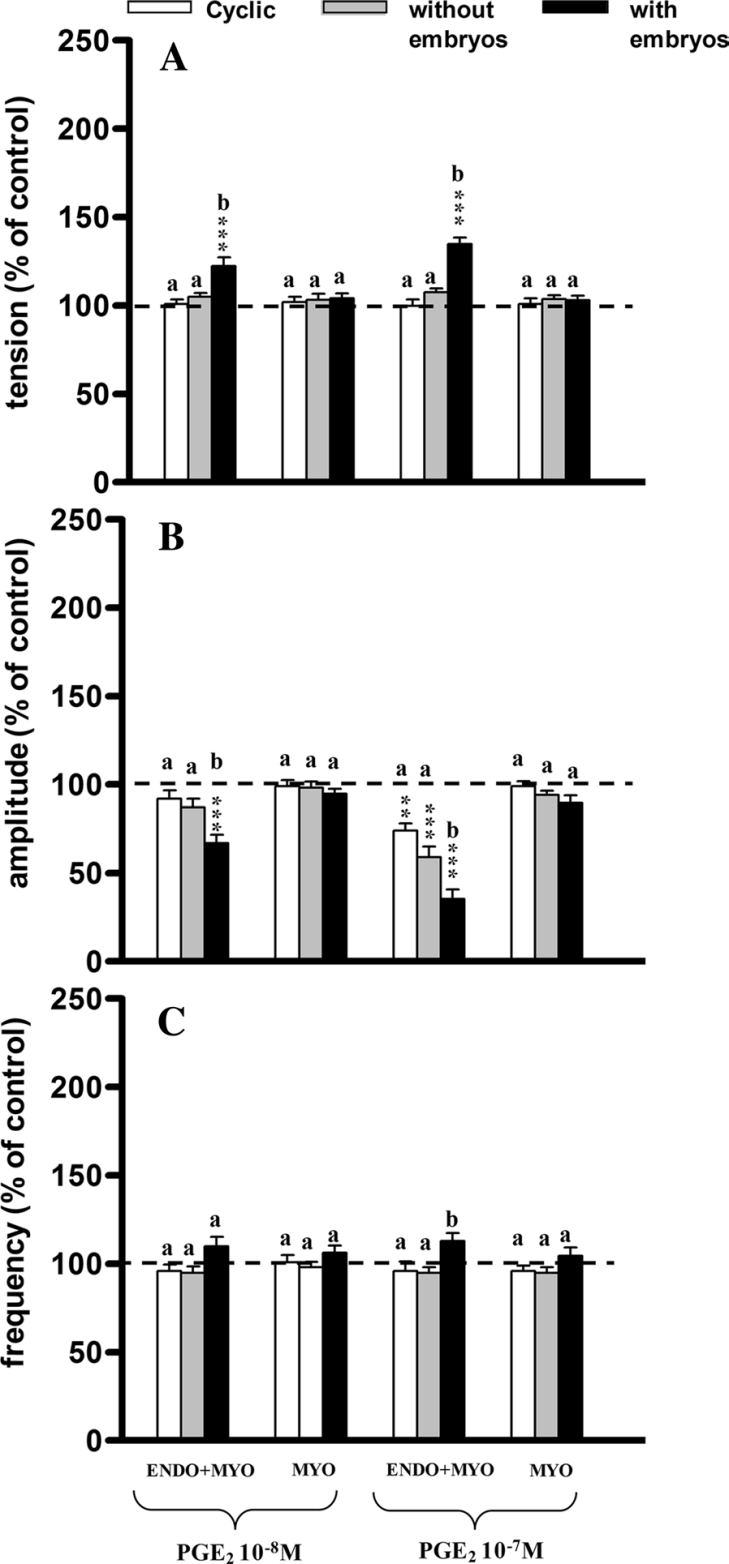
Influence of prostaglandin E_2_ (PGE_2_) on the tension (**A**) amplitude (**B**) and frequency (**C**) of the contraction of the porcine endometrium/myometrium (ENDO+MYO) and myometrium (MYO) strips collected on days 12–14 of the oestrous cycle (cyclic) or after first insemination) (groups without embryos and with embryos). Values (mean ± SD) represents five uterine horns examined in two strips from each one and are expressed as a percentage of changes in the contractile activity before the treatment. **P < 0.01, ***P < 0.001 as compared to the contractile activity before the treatment; *a*, *b*, *c* indicates differences between groups (cyclic, without embryos and with embryos)

At both doses PGE_2_ caused a significant increase (*P* < 0.001) in tension only in the ENDO/MYO strips from group IIb as compared to the pretreatment period (Fig. 6A), as well as compared to groups I and IIa (*P* < 0.01−*P* < 0.001).

In the ENDO/MYO strips PGE_2_ at the smaller dose decreased the amplitude in group IIb (*P* < 0.001), whereas it decreased the amplitude at the higher dose in all groups (*P* < 0.01−*P* < 0.001) as compared to the pretreatment period (Fig. 6B). Significantly lower amplitude was observed in the ENDO/MYO in group IIb as compared to group I (*P* < 0.01) and group IIa (*P* < 0.05).

The frequency of contractions did not change significantly in any of the analysed groups as compared to the pretreatment period (Fig. 6C). The frequency of contractions significantly (*P* < 0.05) increased in the ENDO/MYO strips of group IIb as compared to groups I and IIa.

## Discussion

The presented results demonstrate the influence of biologically active substances on smooth muscle contractility collected from cyclic gilts and uterine horns with or without embryos. The results indicate that in the uterus with embryos contractile activity is increased in comparison to the uterus with not embryos. These differences were clearer when compared to contractility in the uterus of cyclic gilts. The experiments in vitro of Pope et al. [25] showed that myometrial contractility increased concomitantly with embryo migration.

In our studies the ACh increased tension in the smooth muscle of the uterus, which was the highest in the strips of pregnant uterus, with a simultaneous decrease in amplitude. Moreover, the frequency of contractions in the strips of the uterus without embryos was higher as compared to the uterine horn with embryos. Our results are consistent with the results of other authors. Kim et al. [26] observed that after administration of carbachol, a muscarinic receptor agonist, the force of contraction of the uterus in pregnant rats was several times higher than in the non-pregnant uterus. Kitazawa et al. [5] found that both exogenous and endogenous ACh cause contraction of the uterine muscle in the pig by the activation of the muscarinic M_3_ receptor. Our results also indicated that in the uterine strips with embryos the highest increase in tension is combined with a higher decrease of amplitude and a lower increase in the frequency of contractions as compared to the other groups examined. This data indicate that the high motor activity in the gravid horn can promote uniform distribution of the multiple embryos in the lumen of the pig uterus.

One of the regulatory mechanisms affecting uterine contractile activity is the stimulation of α- and β-adrenergic receptors by catecholamines such as NA. In our study, we observed that NA had no significant effect on muscle tension in any group during the 10-min observation period, although in some strips (in each group) a short-term increase in tension after its administration was seen. However, in comparison to the group I NA had a significant influence on the changes of amplitude and frequency of contractions in group IIb, which suggests that the presence of the embryo in the uterus affects the regulation of contractile activity. It is suggested that inhibition of uterine contractile activity is due to a prevalence of β-adrenergic over the α-adrenergic receptors, and may be related to hormonal changes, particularly in the levels of ovarian steroid hormones, which affect the concentration and distribution of the population of muscarinic and adrenergic receptors [27–30]. Rexroad and Guthrie [31] suggested that a reduction in the number of α-adrenergic receptors in the early days of pregnancy may be associated with the migration and implantation of embryos. In this way, the embryos may affect the uterus, e.g., causing changes in the expression of maternal factors. Furthermore, it was observed that embryos may physically affect the uterus by, e.g., changing the expression of genes responsible for the release of biologically active substances [32].

In our study the greatest increase in the tension and frequency of contractions after OT administration was observed in group IIb. Franczak and Bogacki [18] using the same pig model showed that embryonic products locally regulate the abundance of OT receptors mRNA in the porcine uterus. Moreover, it has been shown that the number of receptors for this peptide at 14–16 days of gestation, compared with days 14–16 of the cycle, is similar in MYO but much higher in ENDO [16]. The role of OT receptors in early pregnancy is not fully understood. It is assumed that the peptide, by interaction with its receptors, affects the secretion of prostaglandins, including PGF_2α_ and PGE_2_.

In our study, PGF_2α_ caused the largest increase in tension and amplitude in group IIb, with a decline in frequency in all groups. The weakest effect among all the tested substances was observed after PGE_2_ treatment. However, in the ENDO/MYO strips of group IIb it increased the tension and frequency and decreased the amplitude of contractions. In the reproductive system prostaglandin F_2α_ and E_2_ participates in the control of many process, including implantation of embryos and causing contractions of the uterus [33]. Dittrich et al. [34] demonstrated that human seminal plasma (consisting among others prostaglandins) has a direct effect on enhancing uterine contractility, which could facilitate sperm transport. It is suggested that prostaglandin secretion disorders during pregnancy are one of the causes of miscarriages and premature births. Currently, it is known that the myometrium secretes more PGE_2_ than PGF_2_α regardless of pregnancy status, and that the myometrium may be an additional important source of luteotrophic PGE_2_ action during early pregnancy [17]. It was also found that PGs may play a significant role in embryo implantation and decidualization of the endometrium [23].

Kitazawa et al. [3], studying the activity of smooth muscle of the uterus of pregnant pig, stated, similarly to our study, that ACh, OT and PGF_2α_ increased contractility in muscle. However, in our study the tension and frequency of contractions were increased, while Kitazawa et al. [3] observed a reduction in the frequency of contractions. This difference may be due to the fact that in our study strips were taken at 12–14 days after first insemination, whereas Kitazawa et al. [3] used tissue from 25–60 days of pregnancy. It is accepted that pregnancy changes the expression of receptors, such as oxytocin receptor [35, 36] α_2_-adrenoceptor [37] and prostanoids receptors [38]. Therefore, the increase in the expression of contractile receptors in the uteri of pregnant pigs was suggested to be another mechanism for the pregnancy-associated increase in the contractile responses.

## Conclusions

Summarizing, ACh, OT, PGF_2α_ and PGE_2_ significantly increased the tension and decreased the amplitude of contraction (except PGF_2α_ whose administration increased the amplitude) in gravid horns compared to non-gravid horns and cyclic gilts. The frequency of contractions was increased in the uterine horn with embryos after the administration of ACh and OT, but this parameter decreased after NA and PGF_2α_, and remained practically unchanged after the administration of PGE_2_. Surprisingly, the contractility of the uterine smooth muscle collected from gravid horns exhibited higher activity than that obtained from non-gravid horns and cyclic gilts. Perhaps this is connected with the time of implantation and uniform distribution of embryos. From the data of this study it seem possible to speculate that the presence of embryos may increase the release of some substances which, by auto and paracrine regulation, affect uterine contractile activity.
